# Computerized intensive insulin dosing can mitigate hypoglycemia and achieve tight glycemic control when glucose measurement is performed frequently and on time

**DOI:** 10.1186/cc8129

**Published:** 2009-10-12

**Authors:** Rattan Juneja, Corbin P Roudebush, Stanley A Nasraway, Adam A Golas, Judith Jacobi, Joni Carroll, Deborah Nelson, Victor J Abad, Samuel J Flanders

**Affiliations:** 1 Division of Endocrinology, Indiana University School of Medicine, 545 Barnhill Drive, EH 421, Indianapolis, IN 46202, USA; 2 Department of Medicine and Clarian Health, Indiana University School of Medicine, 545 Barnhill Drive, EH 421, Indianapolis, IN 46202, USA; 3 Department of Surgery, Tufts Medical Center, Tufts University School of Medicine, 750 Washington Street, NEMC Box 4630, Boston, MA 02111, USA; 4 Methodist Hospital/Clarian Health, 1701 N. Senate Blvd., Indianapolis, IN 46202, USA; 5 Medical Quality, Clarian Health, ERC 6102, 1701 N. Senate Blvd., Indianapolis, IN 46202, USA; 6 The Epsilon Group Virginia LLC, 615 Woodbrook Drive, Charlottesville, VA 22901, USA; 7 William Beaumont Hospital, 3601 W 13 Mile Road, Royal Oak, MI 48073-9952, USA

## Abstract

**Introduction:**

Control of blood glucose (BG) in critically ill patients is considered important, but is difficult to achieve, and often associated with increased risk of hypoglycemia. We examined the use of a computerized insulin dosing algorithm to manage hyperglycemia with particular attention to frequency and conditions surrounding hypoglycemic events.

**Methods:**

This is a retrospective analysis of adult patients with hyperglycemia receiving intravenous (IV) insulin therapy from March 2006 to December 2007 in the intensive care units of 2 tertiary care teaching hospitals. Patients placed on a glycemic control protocol using the Clarian GlucoStabilizer™ IV insulin dosing calculator with a target range of 4.4-6.1 mmol/L were analyzed. Metrics included time to target, time in target, mean blood glucose ± standard deviation, % measures in hypoglycemic ranges <3.9 mmol/L, per-patient hypoglycemia, and BG testing interval.

**Results:**

4,588 ICU patients were treated with the GlucoStabilizer to a BG target range of 4.4-6.1 mmol/L. We observed 254 severe hypoglycemia episodes (BG <2.2 mmol/L) in 195 patients, representing 0.1% of all measurements, and in 4.25% of patients or 0.6 episodes per 1000 hours on insulin infusion. The most common contributing cause for hypoglycemia was measurement delay (n = 170, 66.9%). The median (interquartile range) time to achieve the target range was 5.9 (3.8 - 8.9) hours. Nearly all (97.5%) of patients achieved target and remained in target 73.4% of the time. The mean BG (± SD) after achieving target was 5.4 (± 0.52) mmol/L. Targeted blood glucose levels were achieved at similar rates with low incidence of severe hypoglycemia in patients with and without diabetes, sepsis, renal, and cardiovascular disease.

**Conclusions:**

Glycemic control to a lower glucose target range can be achieved using a computerized insulin dosing protocol. With particular attention to timely measurement and adjustment of insulin doses the risk of hypoglycemia experienced can be minimized.

## Introduction

Hyperglycemia is a recognized adverse factor for intensive care unit (ICU) outcomes [1,2]. The landmark study by van den Berghe and colleagues in 2001 provided evidence for a causal link between tight glycemic control and reduced morbidity and mortality in a surgical ICU population [3]. Observational studies outside of clinical trials supported these results, finding improved outcomes after intensive insulin therapy to manage hyperglycemia in the critically ill patient [4-6]. Based on these results and subsequent published guidelines [7,8], hospitals increasingly adopted glycemic control programs, despite controversy regarding how best to use continuous insulin therapy to normalize glucose, the optimal target ranges for improved outcomes and patient populations that most benefit.

Attempts to replicate these early studies have raised concerns about the safety of 'tight' glycemic control protocols. Several large randomized controlled trials were stopped due to unacceptably high rates of severe hypoglycemia (blood glucose (BG) <2.2 mmol/L), 9.8% of patients in the Glucontrol study [9] and 17.0% of the tight control group in the Efficacy of Volume Substitution and Insulin Therapy in Severe Sepsis (VISEP) study [10]. Similarly, 18.7% of the intervention group in the Leuven II medical ICU study experienced severe hypoglycemia, increasing to 25% among patients with ICU stays of 5 days or longer [11]. Most recently, intensive glucose control in the Normoglycemia in Intensive Care Evaluation and Survival Using Glucose Algorithm Regulation (NICE-SUGAR) trial [12] was associated with a 14-fold increase in severe hypoglycemia (6.8%) compared with the moderate glucose control group (0.5%; *P *< 0.001). Subsequently, two meta-analyses also demonstrated that severe hypoglycemia increased the likelihood of death six-fold [13,14]. This overarching concern for hypoglycemia has resulted in a call for more measured, less aggressive glycemic control [13-15], and higher target BG ranges (6.1 to 7.7 mmol/L and 7.8 to 10.0 mmol/L) with recommendations against BG lower than 6.1 mmol/L [15].

These recent results have left clinicians sitting on the horns of the dilemma; how to achieve and maintain glucose control without increasing the risk of hypoglycemia [16]. One reason for this dilemma might be that intravenous (IV) insulin protocols have been designed to lower BG in order to achieve a 'normal' or 'optimal' BG target range, without consideration for their tendency to cause hypoglycemia. Indeed, the literature on manual and computerized protocols reports wide variation in performance in terms of patients reaching target and hypoglycemia rates varying from 4.6% to over 25.0% [17-20]. Moreover, the variety of methods used to measure BG (and their relative accuracy), and the metrics used to define and report hypoglycemia make it challenging to ascertain the actual risk of hypoglycemia with any degree of certainty [21].

On one hand, paper protocols require manual calculation and documentation based on a single BG measure, without consideration of the patient's insulin sensitivity and response to previous dosing. On the other hand, computerized applications, which enable rapid, complex calculations for recommended insulin infusion rates, have demonstrated superior overall efficacy and safety in some reports [22-26], and failed to improve glycemic control or reduce hypoglycemia in others [27,28] when compared with manual protocols.

We previously reported our experience with a computerized IV insulin protocol, the GlucoStabilizer™ achieving BG targets of 4.4 to 6.1 mmol/L in 61.0% of patients with minimal hypoglycemia (<2.8 mmol/L, 4.25%) [29]. Given the concerns surrounding hypoglycemia with intensive insulin therapy, we examine herein, factors contributing to hypoglycemia in the context of the overall performance metrics of the GlucoStabilizer.

## Materials and methods

This study was performed with the approval of the Indiana University human subjects investigational review board. Based on the retrospective and non-interventional nature of this research, patient consent was not required and was waived. In this study, data were analyzed for adult patients with hyperglycemia treated with the GlucoStabilizer (Medical Decisions Network, Charlottesville, VA, USA) to a target range of 4.4 to 6.1 mmol/L in ICUs from March 2006 to December 2007. The ICUs were large, with 30 and 32 beds available to both medical and surgical patient populations. Illness severity scores were not available for these analyses. Average length of stay was 5.5 days, and patient care required a high nurse to patient ratio (1:2 respectively). Hourly BG measurements were most frequently obtained by fingerstick capillary sampling; however, venous and arterial sampling with point-of-care glucometer, blood gas analyzer and central laboratory measurement were also included, reflecting a real-world clinical context.

Use of the GlucoStabilizer has been previously described in detail [29]. In brief, when a patient BG value is entered, the program calculates an initial insulin infusion rate in units/hour using (BG in mg/dL - 60) × multiplier, set at an initial default of 0.02, (an insulin sensitivity factor) [30]. The BG target range set to 80 to 110 mg/dL (4.4 to 6.1 mmol/L), testing interval set to 60 minutes, and reminder alarms set for 55 minutes were preprogrammed. In the event of hypoglycemia (BG <3.9 mmol/L), the software reverts to a hypoglycemia recovery mode and calculates an appropriate dose of D50W = (100 - BG in mg/dL) × 0.4 mL) to be given IV. An audible alarm alerts the nurse to a scheduled BG check and also every 15 minutes until recovery from the hypoglycemic event to the target BG range. All drip run information and insulin doses are electronically saved in the GlucoStabilizer database.

In the absence of generally accepted performance metrics for IV insulin protocols [21], we evaluated the safety and efficacy of our experience with the GlucoStabilizer using standardized methods to calculate (1) time to target BG; (2) time BG remained in target range of 4.4 to 6.1 mmol/L; (3) rate of hypoglycemia using several metrics including: the proportion of episodes overall (n, % for BG <2.2, <2.8, <3.3, <3.9 mmol/L), percentage of patients experiencing at least one episode of BG less than 2.2 mmol/L, the number of events per patient and the number of events standardized to 1000 drip run hours, time to hypoglycemia episode and time spent in hypoglycemia. We used at least one episode of BG less than 2.2 mmol/L as a measure of critical and severe hypoglycemia, to correspond with the most common definition of serious hypoglycemia reported in the literature.

We used a 'patient drip run' as the unit of analysis when calculating time to achieving target BG range, percentage of time within target range, and incidence of hypoglycemia. A patient drip run starts with entry of a patient's identifying data and initial BG into the GlucoStabilizer program. IV insulin drip runs are generally initiated after two BGs above 7.2 mmol/L determined by either point-of-care or laboratory measurements. For analysis purposes, a drip run is considered complete when there is a gap of six hours or more between successive BG measurements. The same patient can thus have multiple drip runs, as might occur when IV insulin is restarted after a period of normal BG followed by later reoccurrence of hyperglycemia. Drip runs started for patients with a baseline BG of 6.1 mmol/L or less were not included in this analysis,

A 'patient time-glucose curve' is used in this analysis as a continuous representation of glucose readings resulting from a drip run over time. This curve is constructed by plotting the discrete set of time and BG pairs as points, with time on the ×-axis and BG on the y-axis. These points are connected by line segments, producing a curve that approximates the patient's BG at any time during the drip run. The percentage of time that a patient was within a particular BG range, for example 4.4 to 6.1 mmol/L, was calculated relative to this curve, as was one-hour BG change. The patient time-glucose curve was constructed for 50 hours' duration (mean drip run length for this patient population).

In addition to analyzing these parameters, we were interested in performance of the software protocol within different disease subgroups. Patient populations for subgroup analysis were identified using *International Classification of Diseases*, 9^th ^Revision, Clinical Modification diagnosis and procedure codes from our inpatient database: sepsis (038.9 and either 995.91 or 995.92), acute myocardial infarction (410.xx, primary diagnosis only, excluding 410.x2 for follow-up treatment), coronary artery bypass graft (36.1x, primary procedure only), all diabetes mellitus (250.xx), type 1 diabetes mellitus (250.x1 or 250.x3), new onset acute renal failure (584.5, 584.61, 584.7, 584.8, or 584.9), chronic kidney disease (585.x), and those on dialysis [38.95].

### Statistical analysis

Insulin drip runs were analyzed using the median (interquartile range [IQR]) measure. Kaplan-Meier time-to-event curves were used to estimate time to achieve target range. Hypoglycemia was examined by using Kaplan-Meyer time-to-event curves to estimate time-to-hypoglycemia, calculated as the amount of time into the drip run when a hypoglycemic BG was first recorded. Descriptive statistics were used to calculate frequency of hypoglycemia events in terms of percent of events, number of events per patient, and events standardized to 1000 drip-run hours. A further analysis to determine the influence of timing of BG measurement on the occurrence of hypoglycemia events was modeled by examining the rate of change between measures as a predictor of hypoglycemia when measurement was delayed. Subgroup comparisons were made using the Chi-squared statistic. All analyses were performed using SPSS 15.0 (SPSS Inc, Chicago, IL, USA).

## Results

During the study period, 4588 ICU patients were treated with the GlucoStabilizer to a target range of 4.4 to 6.1 mmol/L. There were a total of 6069 drip runs recorded in the GlucoStabilizer database for these patients. Runs where the starting BG was 6.1 mmol/L or less were excluded leaving 5456 runs for this analysis. The median (IQR) drip run length was 40.3 (19.2 to 83.0) hours.

### Time to target and time within target

**Figure 1:**
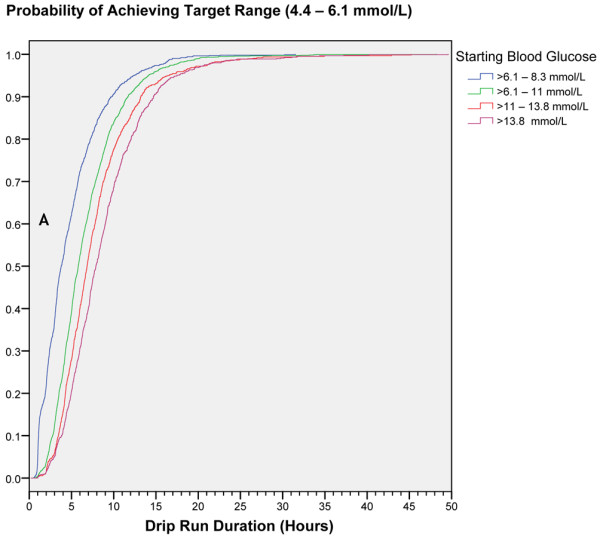
Time to achieve target range for starting blood glucose. Kaplan-Meier time-to-event curves for the time to achieve target range 4.4 to 6.1 mmol/L, for starting blood glucose ranges >6.1 to 8.3 mmol/L, >8.3 to 11 mmol/L, >11 to 13.8 mmol/L, and >13.8 mmol/L.

**Figure 2:**
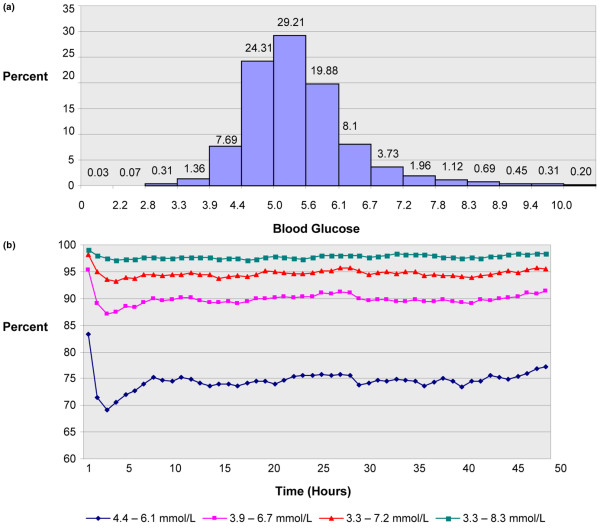
Proportion of measures reaching target and remaining in target. Percentage of measures in selected blood glucose ranges after target range of 4.4 to 6.1 mmol/L achieved. **(a) **Percentage of measures in selected ranges using 0.55 mmol/L intervals; **(b) **Percentage of time blood glucose measures were in target for ranges 4.4 to 6.1, 3.3 to 6.7, 3.3 to 7.2, and 3.3 to 8.3 mmol/L for the first 50 hours after target range achieved.

The median (IQR) time to achieve target range was 5.9 (3.8 to 8.9) hours. The median times to achieve target range were longer for higher initial BG; times to target (IQR) for baseline BG in ranges of more than 6.1 to 8.3, more than 8.3 to 11.1, more than 11.1 to 13.8, and more than 13.8 mmol/L were 3.8 (2.1 to 6.4), 5.8 (4.0 to 8.6), 6.8 (4.7 to 9.6), and 7.9 (5.4 to 11.0) hours, respectively. Kaplan-Meier time-to-event curves for the time to achieve target range demonstrate this dependence on the baseline BG (Figure 1). Almost all patients (97.5%) achieved the target range. Mean BG (± standard deviation (SD)) after achieving target was 5.4 (± 0.52) mmol/L. After reaching target, patients remained in target 4.4 to 6.1 mmol/L, 73.4% of the time, and in the expanded ranges of 3.9 to 6.6 mmol/L and 3.9 to 8.3 mmol/L, 89.2% and 95.9% of the time respectively, over 50 hours of the drip run. (Figures 2a and 2b)

### Hypoglycemia

**Figure 3:**
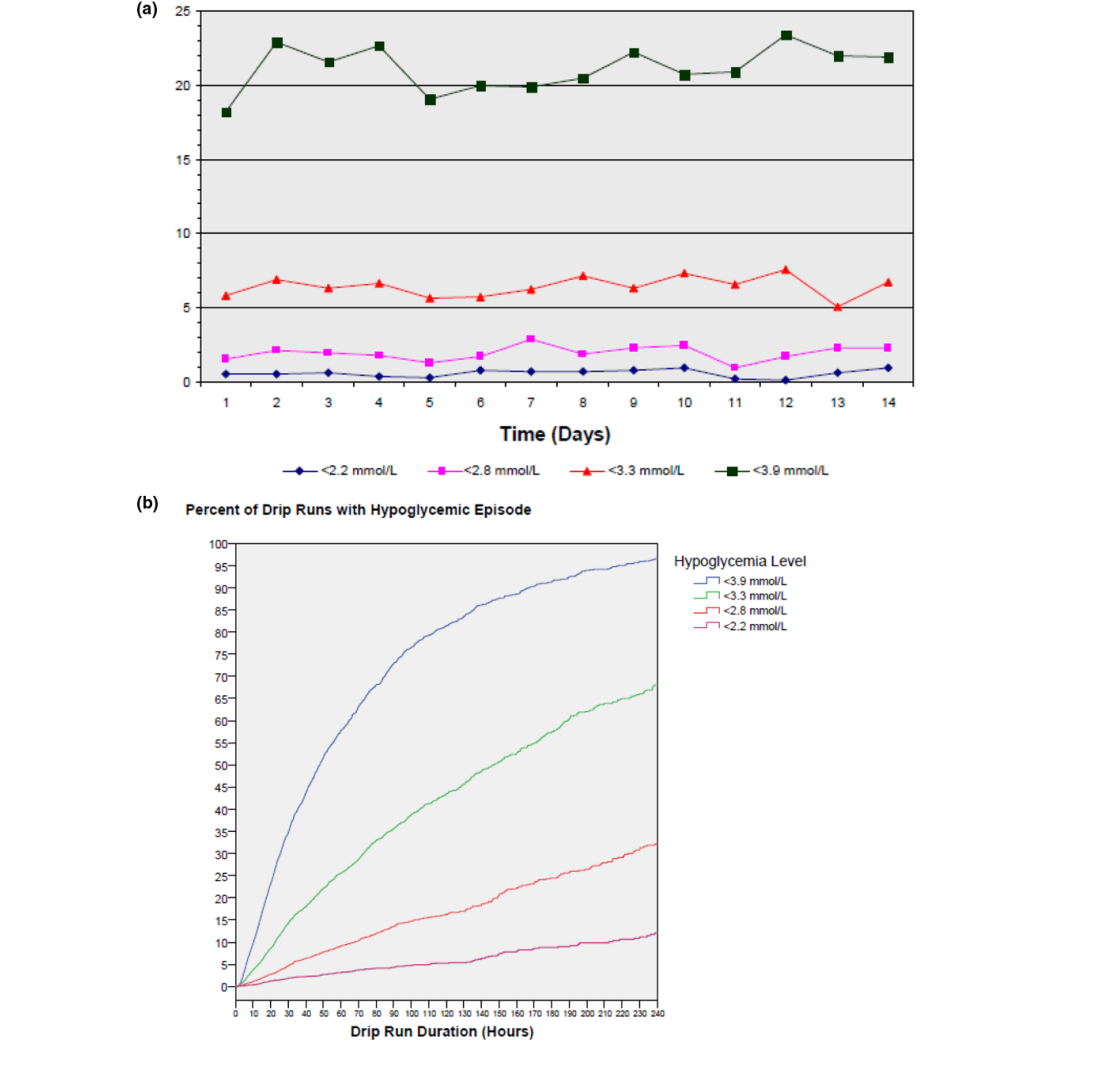
Hypoglycemia incidence. **(a) **Number of hypoglycemic episodes per 1000 hours over the first 14 days; **(b) **Kaplan-Meier time-to-event curves for the times to hypoglycemia <2.2, <2.8, <3.3, and <3.9 mmol/L.

A 4.25% proportion of patients experienced at least one episode of BG less than 2.2 mmol/L. We observed 254 episodes of severe hypoglycemia in 195 patients, with 32 patients experiencing more than one event. Overall, 0.1% of the 289,289 BGs in the database were less than 2.2 mmol/L and corresponding rates for less than 2.8, less than 3.3 and less than 3.9 mmol/L were 0.07%, 0.31% and 1.36%, respectively (Figure 2a). Over the first 14 days of IV insulin therapy, the hypoglycemia incidence standardized to 1000 hours are shown in Figure 3a; with rates of 0.60, 1.89, 6.35, 20.5 for the increments of hypoglycemia. The Kaplan-Meier time-to-event curves for the time to hypoglycemia for less than 2.2, less than 2.8, less than 3.3, and less than 3.9 mmol/L illustrate that the incidence of hypoglycemia increased with the duration of the drip (Figure 3b). For drip runs lasting 24, 48, 72, 96, and 120 hours, the percentage with at least one episode of severe hypoglycemia (BG <2.2 mmol/L) was 1.1, 2.5, 3.8, 4.7, and 5.4% respectively.

We further examined the 254 episodes (n = 195 patients) of severe hypoglycemia (BG <2.2 mmol/L) in the 4588 patients. Assuming that BG changes at a constant rate between measurements in response to a constant insulin dose, we used the following model to examine time to hypoglycemia and time spent in hypoglycemia: for each hypoglycemic measure, we assumed a constant decrease of 0.05 mmol/L/min from the previous BG measure, based on usual protocol performance. For example, if a measure of 6.1 mmol/L at 1200 hours was followed by a measure of 1.7 mmol/L at 1320 hours (80 minutes later); using our model, the level of 2.2 mmol/L would be reached in 70 minutes, that is at 1310 hours. An on-time measurement at 1255 hours (when estimated BG would have been 3.0 mmol/L), would have triggered hypoglycemia recovery mode by the software and the avoidance of the severe hypoglycemic event. Using this model we found that in 170 (66.9%) of the 254 severe hypoglycemia episodes, the duration of measurement delay exceeded the estimated time to hypoglycemia, suggesting that delayed measurement by 12 minutes (median 21.8 minutes, IQR 12.2 to 29.0 minutes) may have contributed to these severe hypoglycemic episodes. In 116 (45.7%) of the severe hypoglycemic episodes, the previously measured BG exceeded 6.1 mmol/L, demonstrating that severe hypoglycemia may also be associated with large, (>3.3 mmol/L/hr decrease) rapid (more than 0.05 mmol/L/min), and unpredictable drops in BG. Two hundred and one (79.1%) episodes were associated with either a previous BG of more than 6.1 mmol/L or a measurement delay that exceeded the estimated time to hypoglycemia and 85 episodes were associated with both. No discernable cause for hypoglycemia occurrence could be determined in 84 patients. In evaluating all the hypoglycemic events, the mean (± SD) amount of time that a patient was severely hypoglycemic before detection was 8.9 ± 7.4 minutes. The time to BG recovery to 2.2 mmol/L was 3.8 ± 6.1 minutes, and time to target recovery of more than 3.9 mmol/L was 28.0 ± 26.2 minutes; with a mean BG of 6.1 ± 2.9 mmol/L at recovery.

**Figure 4:**
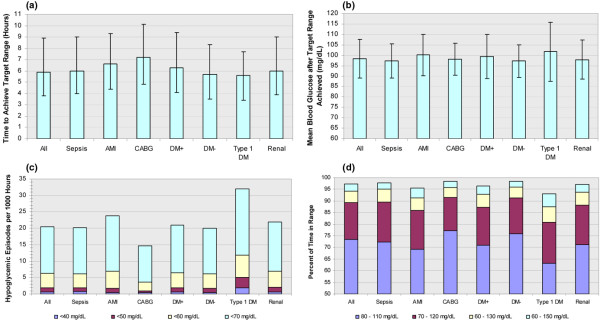
GlucoStabilizer management of all patients, compared with those with AMI, CABG, diabetes, and renal failure. **(a) **Time to achieve target range, hours, median and interquartile range; **(b) **Mean blood glucose after target range achieved, mean and standard deviation; **(c) **Number of hypoglycemic episodes per 1000 hours; **(d) **Percent of time in ranges 4.4 to 6.1, 3.9 to 6.7, 3.9 to 7.2, and 3.9 to 8.3 mmol/L after target range achieved. All = all patients (5456 drip runs); Sepsis = (658 drip runs); AMI = acute myocardial infarction (160 drip runs); CABG = coronary artery bypass graft (444 drip runs); DM+ = with diabetes (2717 drip runs); DM- = without diabetes (2647 drip runs); Type 1 DM = type 1 diabetes (126 drip runs); Renal = new onset acute renal failure, chronic kidney disease, or on dialysis (2207 drip runs). The value in mmol/L can be calculated by multiplying the mg/dL value by 0.05551.

Finally, GlucoStabilizer performance was found to be comparably effective in several critically ill patient populations, with similar time-to-target and time-within-target durations in patients with and without diabetes, and with admission diagnosis of sepsis, acute myocardial infarction, coronary artery bypass graft, or renal disease. Hypoglycemia rates were also low in all patient subtypes studied with the highest rate seen in patients with type 1 diabetes (Figure 4).

## Discussion

The challenge of inpatient hyperglycemia management is to find a balance between two disparate and competing goals; that of correcting hyperglycemia while minimizing and preventing hypoglycemia. When insulin is administered, hypoglycemia is a foreseeable consequence, and more likely to occur with more aggressive and narrow BG target ranges. In contrast to some of the commonly used protocols, the GlucoStabilizer achieved target more often (97.5%) for longer duration, with a comparable incidence of severe hypoglycemia (4.25% per patient, and 0.1% per measure). Additionally, we found the hypoglycemia rate would be considerably less (2.0% per patient) with timely BG measurement. The Leuven paper-based protocol [3], using a target range of 4.4 to 6.1 mmol/L showed ability to reach a mean BG of 5.7 ± 1.1 mmol/L and a significant improvement in mortality and morbidity for surgical ICU patients with a severe hypoglycemia rate of 5.1% (any BG ≤ 2.2 mmol/L). The Leuven protocol and target ranges were subsequently implemented in two other large multicenter randomized trials that were stopped prematurely due to excessive, significant hypoglycemia [9,10]. In VISEP [10] the hypoglycemia rate was 17.0% compared with 4.1% in the control group, while in GLUCONTROL [9] the incidence of hypoglycemia (BG <2.2 mmol/L) was 9.8% in the intensive group compared with 2.7% in the control group. It is of interest that these studies utilized the same protocol, yet realized such different rates of hypoglycemia, demonstrating that factors outside of the protocol rather than the glucose target may significantly influence glucose control and hypoglycemia.

Regarding other protocols, in a study of cardiac surgery patients with diabetes, 61% of patients achieved the glucose target range of 4.4 to 8.3 mmol/L while on continuous insulin infusion based on the Portland protocol (paper-based) with a 7.1% hypoglycemia rate (BG <2.2 mmol/L) [4]. In contrast, a very low incidence of hypoglycemia was reported in a study using the Yale protocol in cardiothoracic ICU and medical ICU patients (0.2% and 0.3%, respectively, with successful glycemic control in 73% and 66% of both populations (target BG range of 4.4 to 7.7 mmol/L). However, hypoglycemia was defined as a BG less than 3.3 mmol/L in these studies [28]. Similarly, 53.9% of all patient measures were in the 4.4 to 6.1 mmol/L target while only 0.1% of measures fell below 4.0 mmol/L in a study using the computer-derived, but paper-based Specialised Relative Insulin Nutrition Tables protocol [29]. Two studies comparing the Model Predictive Control Algorithm to the routine paper glucose management protocols, found improved glycemic control based on lower mean BG achieved, and longer time in target range and low rates of hypoglycemia [25,31,32]. These studies were not included in a review of the relative risk of hypoglycemia with intensive insulin therapy in the ICU [13] which reported the incidence of hypoglycemia and outcomes among the patients treated with these various protocols of various targets and hypoglycemia definitions. Hypoglycemia incidence ranged from 5.0% to 18.7% in tight glucose target groups, and found a significantly increased overall risk of hypoglycemia (13.7% vs. 2.5%; relative risk 5.13; 95% confidence interval 4.10 to 6.43) with insulin treatment to lower glycemic targets [13]. An updated meta-analysis that included the NICE-SUGAR results again found a six-fold increased risk of severe hypoglycemia among patients given intensive insulin therapy compared with controls, with little examination of the protocols represented [14]. In fact, on examination of the protocols included, it appears all are clinically derived, and there is little that differentiates the basic elements of these protocols except their reported success or failure in various populations and settings.

When evaluating reports of experience with computerized insulin infusion protocols, some have shown improved glycemic control with reduced time-to-target, longer time-in-target and lower rates of hypoglycemia [20,22-35]. Most of these protocols have two attributes in common; an insulin dose calculator that uses a current and previous BG value (considering the insulin sensitivity) and a recommendation for the timing of the next BG measure. Protocol compliance can exceed 90%, and achieve better glucose control when compared with paper-based protocol [34]. But, even when these factors are in place, it may be difficult to achieve low BG targets without hypoglycemia. It must be noted that although the NICE-SUGAR web-based insulin dose calculation protocol was standardized across 42 centers there was a high rate of protocol deviation. In an interim safety analysis of the first 100 hypoglycemic events occurring in the study, 8.0% of patients treated in the intensive glycemic control arm experienced severe hypoglycemia (BG <2.2 mmol/L) versus 0.3% in the moderately controlled group. Adjudicated causes were reported to be clinician error (failure to follow the computerized treatment algorithm and infrequent BG monitoring in 37%, decreased nutritional intake 24%, pre-terminal state 8%, spurious measurement error 16%, and other miscellaneous causes in 15% [34-36] Additionally, BG measures were taken at various intervals (one to four-hour intervals) with no reminder system to support timely BG testing. Many protocols lack the audible reminders to perform timely BG measurement and insulin dose adjustment-critical factors for safe and effective glycemic control, as demonstrated in our analysis. Additionally, ameliorating factors that predispose to hypoglycemia [37-39] and management of rapid fluctuations in BG levels with prompt, frequent, accurate and timely glucose measurements are external factors that contribute to the success or failure of any protocol [40].

So although many reports and numerous editorials have called tight glycemic control strategies into question citing the inherent risk of hypoglycemia and association with mortality, they have largely overlooked performance characteristics of the IV insulin protocols used in the studies. Protocol comparisons have shown that different BG targets were used, there were differences in study populations, differences in definitions of hypoglycemia, all of which contributed to wide disparities in the performances of IV insulin protocols thereby precluding reasonable comparisons in efficacy of therapy and outcomes [13,14,17-20]. As such, the study outcomes may more reflect protocol compliance than protocol performance and their influence cannot be isolated.

In contrast, the GlucoStabilizer studied herein demonstrated a high likelihood of achieving target BG with a comparably low incidence of hypoglycemic events across our large ICU population. Additionally, nearly 90% of patients not only achieved BG control within the range of 3.9 to 6.7 mmol/L but also remained in that range 96% of the time demonstrating that the GlucoStabilizer effectively and safely controls BG. And, finally, we found that the GlucoStabilizer performed consistently among different critically ill patient populations.

However, even in our environment, where the testing interval is one hour, delays in BG measurement were associated with hypoglycemic episodes, ultimately accounting for 67% of observed severe hypoglycemia. In the high-stress environment of an ICU, it is not uncommon that a scheduled BG test is delayed, despite the warning provided by audible alarms from the GlucoStabilizer. Given the rapid action of IV insulin and an aggressive target range, a delay of even eight minutes can result in an episode of hypoglycemia. Additionally, we observed large unpredictable drops in BG within the one hour testing frequency in some patients who experienced severe hypoglycemia. Our examination of hypoglycemic events and their relation to timing of BG measurement is an important new understanding of the causes of hypoglycemia, particularly since hypoglycemia at a level less than 2.2 mmol/L could be independently associated with increased risk of mortality [41]. Our data would further argue that while the occurrence of severe hypoglycemia is a known risk associated with IV insulin, especially with lower glucose targets, the risk is likely compounded with any protocol that can be difficult to use with consistency. This is especially so in a busy ICU setting where critically ill patients and their metabolic demands can change with little notice. All of these factors contribute to inadvertent and unintentional errors and delays in BG testing that may result in hypoglycemia and illustrate the limitations of current measurement technologies when used to achieve a strict glycemic target.

A major limitation of our study is its non-randomized, retrospective nature, which does not allow for direct comparison and unequivocal evidence that our computerized system is superior to a paper IV insulin protocol. However, this system as well as other computerized dosing calculators has demonstrated that they are able to achieve tight glucose targets and maintain patients in narrow therapeutic ranges of BG, with low rates of hypoglycemia in published reports and clinical experience. The methodologies used to examine hypoglycemic events in this paper could be useful in evaluating other protocols and such events in other randomized clinical trials where IV insulin is utilized.

The authors acknowledge several significant limitations of this investigation. Illness severity scores were not available for these analyses; however, average length of stay was 5.5 days, and patient care required a high nurse to patient ratio (1:2 respectively). Also, hourly blood glucose measurements were most frequently obtained by fingerstick capillary measures, with some venous and arterial sampling using point-of-care glucose meter, blood gas analyzer and central laboratory measurements. It is unknown how our results would differ if BG measurement methodologies were controlled for in this analysis. This study reflects a real-world clinical context, and the influence of accuracy of the BG values is a subject of further study. Finally, we have not presented the relation of patient outcomes with the various indices of glycemic control, and plan to include that in future analysis.

## Conclusions

Performance characteristics of insulin dosing protocols cannot be overlooked when evaluating the evidence for tight glycemic control and resulting hypoglycemia. Factors including timely, frequent, accurate BG measurement and treatment with correct and prompt insulin dose adjustment contribute to safe and effective glycemic control to any target. Our results show that a computerized IV insulin protocol can be successfully implemented on a large scale in multiple ICUs in a variety of patient conditions. We found a low rate of hypoglycemia, compared with reports of other protocols, even with the vast majority of patients treated to an aggressive target range of 4.4 to 6.1 mmol/L reaching target and remaining in target. Delays in the timing of repeat BG measurements of more than 12 minutes were found to be an important contributor to 67% of hypoglycemic events.

## Key messages

• Insulin dosing algorithms designed to manage BG levels have been implemented with varying degrees of success in targeting euglycemia and are often associated with high rates of hypoglycemia

• Frequent (hourly) monitoring of BG to facilitate timely insulin dose adjustment is necessary in hypoglycemia prevention, and measurement delay is often associated with increased risk of severe and serious hypoglycemia

• Rapid drop in BG (>3.3 mmol/hour) combined with measurement delay greatly increases the risk of severe and serious hypoglycemia

• Computerized IV insulin dosing can achieve and maintain glycemic control in critically ill patients with low risk of hypoglycemia when BG measurements are performed frequently and on time, thereby facilitating timely insulin dose adjustment.

• Future randomized trials of inpatient glycemic management should employ protocols that have undergone rigorous evaluation and are proven to have low rates of hypoglycemia with high rates of demonstrated protocol compliance to limit the potential confounding factors that predispose to severe hypoglycemia and inpatient mortality.

## Abbreviations

BG: blood glucose; ICU: intensive care unit; IQR: interquartile range; IV: intravenous; NICE SUGAR: The Normoglycemia in Intensive Care Evaluation and Survival Using Glucose Algorithm Regulation; SD: standard deviation; VISEP: Volume Substitution and Insulin Therapy in Severe Sepsis.

## Competing interests

RJ and CPR (deceased) receives royalties from the sale of the CGS dosing tool and RJ is a shareholder for Diabetes Innovations, LLC, a consultancy company that gets paid the royalties from the sale of the CGS dosing tool indicated above. SJF receives royalties from sales of the GlucoStabilizer software and Clarian Health Partners was assigned the patent rights for the Glucostabilizer software. SAN is Clinical consultant for Medical Automation Systems, Echo Therapeutics, Optiscan. JC receives clinical consultant fees paid by Medical Automation Systems who owns license for and markets the IV GlucoStabilizer insulin computer program. AAG, DN, JJ, and VJA have no competing interests.

## Authors' contributions

RJ, CPR, SAN, JJ, and SJF made substantial contributions to the conception and design of the study, and have been involved in drafting and revising the manuscript for critically important content. AAG, JC, and DN have been involved in the acquisition of data and description of the processes involved in glycemic management. VJA is a statistician, and provided analysis and interpretation of the data. All authors have given final approval of this version of the manuscript.
